# Renal cell carcinoma growing into the renal pelvis and mimicking transitional cell carcinoma: A case report and literature review

**DOI:** 10.3892/ol.2015.2898

**Published:** 2015-01-26

**Authors:** YIFAN LI, YU DING, DUQUN CHEN, ZUHU YU, YAOTING GUI, SHANGQI YANG, YONGQING LAI

**Affiliations:** 1 Department of Graduate Medicine, Anhui Medical University, Hefei, Anhui 230032, P.R. China; 2 Department of Urology, Peking University Shenzhen Hospital, Shenzhen, Guangdong 518036, P.R. China; 3 The Guangdong and Shenzhen Key Laboratory of Male Reproductive Medicine and Genetics, Peking University Shenzhen Hospital, Institute of Urology of Shenzhen PKU-HKUST Medical Center, Shenzhen, Guangdong 518036, P.R. China

**Keywords:** renal cell carcinoma, transitional cell carcinoma, computed tomography, ureteroscopy

## Abstract

Renal cell carcinoma (RCC) originated from parenchyma and the majority of malignancies originating in the renal pelvis are transitional cell carcinoma (TCC). In the present study, a rare case of RCC growing into the renal pelvis and mimicking TCC in medical imaging is reported. The preoperative differentiation between RCC and TCC is important in order to identify the type of surgical treatment required: Nephrectomy or ureteronephrectomy. The role of ureteroscopy and biopsy is emphasized in the accurate preoperative diagnosis of a renal pelvic mass. Thus, the present study provided fundamental evidence for the pathogenesis of RCC with pelvic extension and challenged the present tumor node metastasis staging system of RCC.

## Introduction

Renal cell carcinoma (RCC) is the most common type of kidney parenchymal cancer (1). Malignant tumors of the renal pelvis constitute only 5% of urinary tract neoplasms and ~90% of pelvic cancer cases are transitional cell carcinoma (TCC) (2). Although the majority of malignancies originating in the renal pelvis are TCC, RCC has a tendency to grow into the renal pelvis. The imaging findings of RCC and TCC in the renal pelvis are nonspecific; therefore, preoperative differentiation between RCC and TCC is important in order to identify the type of surgical treatment required: Nephrectomy or ureteronephrectomy (3–5). The present study presents a rare case of an RCC growing into the renal pelvis, in which TCC of the renal pelvis was not able to be distinguished using contrast enhanced computed tomography (CT) or CT urography (CTU). Due to the uncertainty of diagnosis, the present study emphasizes the role of ureteroscopy and biopsy in accurate preoperative diagnosis and the theoretical importance of the present case. Written informed consent was obtained from the patient.

## Case report

**Figure 1:**
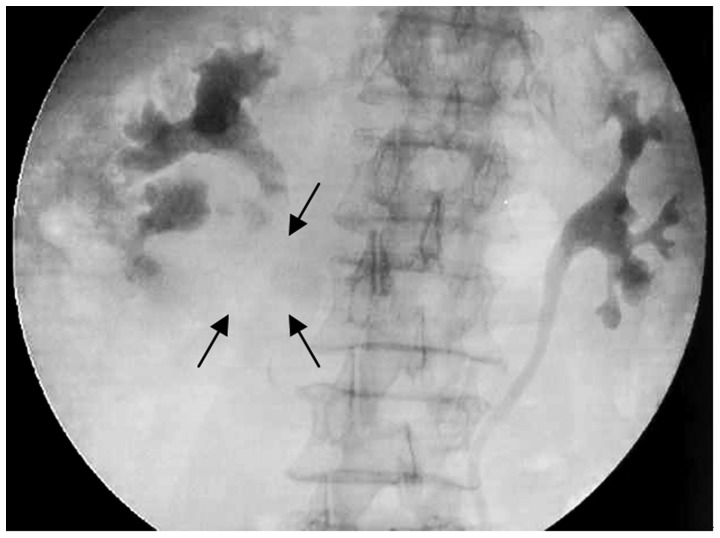
Intravenous pyelogram indicating obstruction of the right renal pelvis by a solid mass (arrows).

In April 2013, a 51-year-old male presented to Peking University Shenzhen Hospital (Shenzhen, China) with the chief complaint of a three-year history of repeated dull pain in the right flank and painless gross hematuria for six months. The patient had undergone two previous surgical procedures of extracorporeal shock wave lithotripsy for renal calculus in 1997 and 2009. Physical examination revealed percussion pain over the right kidney region and laboratory analysis identified a marginal increase in alanine aminotransferase (59.8 U/l; normal range, 5.0–40.0 U/l) and creatine (118.5 μmol/l; normal range, 62–115 μmol/l) levels. In addition, an abundance of red blood cells (2,103 cells/μl; normal range, 0–12 cells/μl) was detected upon urinalysis, while urine cytology identified the presence of inflammatory cells but no atypical cells. Intravenous pyelography (IVP) was performed, demonstrating an obstruction to the right renal pelvis and, therefore, no visualization of the later ureter (Fig. 1). A right kidney tumor was suspected based on the results of a dynamic CT scan, which revealed a marginally-contrasted space-occupying lesion in the right kidney. Furthermore, ureteroscopy verified the presence of a solid intraluminal mass (0.6×0.8 cm), spreading into the upper urinary tract in close proximity to the renal pelvis. The renal pelvic masses exhibited wide bases with high central densities and a brittle surface, which bled easily. A ureteroscopic biopsy was performed prior to placing a double-J stent in the right ureter, which was histologically and cytogenetically analyzed to determine a diagnosis of clear-cell RCC. No apparent metastases to the lung, liver, adrenal glands or lymph nodes were observed.

**Figure 2:**
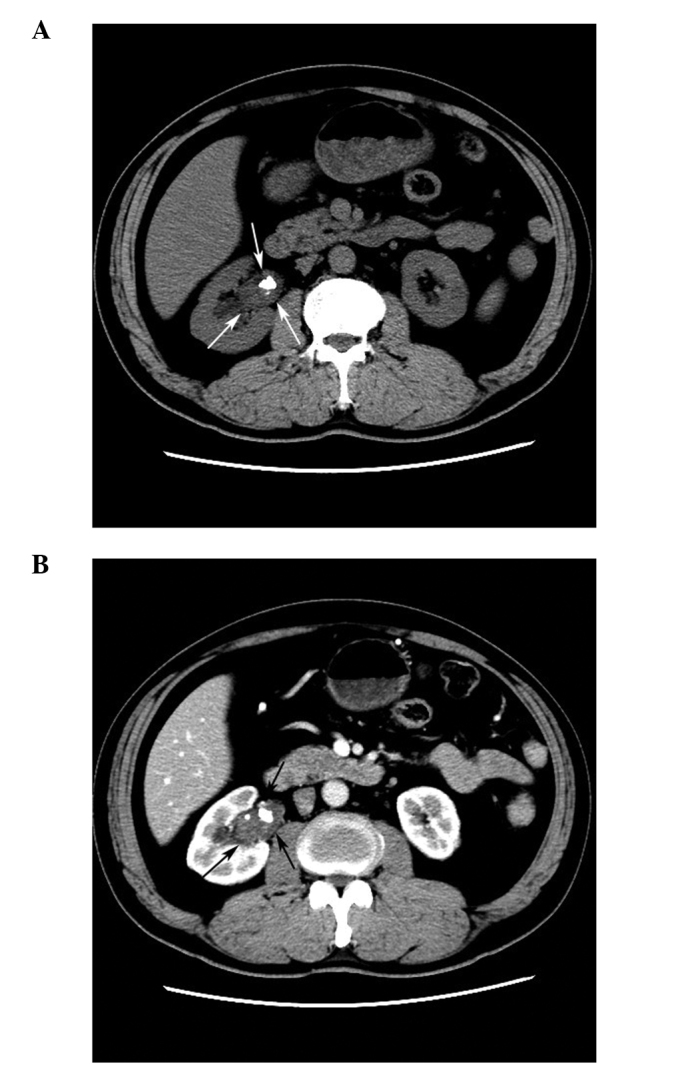
(A) Plain computed tomography (CT) scan. White arrows indicate a 2.0×1.6×3.4-cm mass with a central high density in the right renal pelvis. (B) Contrast-enhanced CT scan. Black arrows indicate that the mass was marginally enhanced in the arterial phase.

Based on the aforementioned results, a diagnosis of a clear-cell RCC of the right kidney was proposed; however, TCC was not be completely excluded. Therefore, to further define the renal pelvic mass, CTU was performed, revealing a 2.0×1.6×3.4 cm papillary right renal pelvic mass without renal parenchymal (Fig. 2A). In the early phase, the tumor presented contrast enhancement and was considered to predominantly consist of TCC (Fig. 2B). Furthermore, the CT value of the tumor was 25 Hounsfield units (HU) in the pre-contrast films and 56 HU in the post-contrast films. However, due to the tentative diagnosis of clear-cell RCC based on the ureteroscopic biopsy, the patient was subjected to open right radical nephrectomy with retention of the ureteral stump. During surgery, frozen section analysis revealed an inflammatory polyp-covered transitional epithelium of mild hyperplasia with blood clots and kidney stones.

Macroscopically, the localized tumor originated in the renal calices of the right kidney lower pole, was attached to the renal pelvis by its base and measured 4.0×1.0×0.5 cm. Microscopically, the tumor was composed of atypical epithelioid cells with a wide distribution, with interstitial edema and infiltration of the focal lymphoid tissue; however, no invasion of the ureteral wall was observed. Immunohistochemically, the tumor exhibited diffuse positive staining for vimentin and P504S, while it was only focally and weakly positive for cytokeratin (CK) 7, cluster of differentiation 10 and Ki-67. In addition, immunostaining for CK20, uroplakin, kidney-specific cadherin and prostate-specific antigen was negative. These findings were in accordance with a diagnosis of RCC; however, it remains unclear whether the tumor was a clear-cell or papillary RCC as the patient declined to undergo further assessment. No malignancy was observed in the local renal pelvic or ureteral mucosa.

The patient was discharged nine days after surgery, following an uncomplicated postsurgical recovery. No evidence of recurrence or residual disease was detected on CT scans during the nine-month follow-up period.

## Discussion

The present study reported the challenging diagnosis of a marginally-enhanced renal pelvic mass that was identified using dynamic CT/CTU. Histological analysis revealed a clear-cell RCC with renal pelvic invasion, which is a rare carcinoma reported only in a small number of cases in the English literature (Table I) (3–9). Due to inadequate preoperative diagnosis of TCC, nephroureterectomy has been performed in a number of previous cases (3,5). However, nephroureterectomy including cuff resection of the bladder wall was performed in one case since frozen section analysis was unable to exclude TCC (8). RCC with renal pelvic extension was possibly mistaken for TCC only based on CT findings or frozen section analysis. Therefore, the results of various diagnostic approaches, particularly contrast enhanced CT/CTU, ureteroscopy and biopsy, in the preoperative diagnosis of such renal pelvic masses should be compared and assessed.

Dynamic CT/CTU has been increasingly performed as an alternative to the traditional method (IVP) for the diagnosis of upper tract lesions, due to its high diagnostic accuracy and favorable comparisons with other imaging techniques (10). However, the principal limitation of CTU is false-positive diagnosis; thus, a renal pelvic mass diagnosed with CTU should be biopsied (guided by ureteroscopy) for histopathological confirmation prior to proceeding with the surgical procedure (10).

An enhanced mass, which may indicate a non-opaque pyelolith, blood clot, lymphoma or, less commonly, RCC and metastasis to the kidney with renal pelvic invasion, has been identified in patients exhibiting painless hematuria using CT/CTU (2,4). Dynamic CT images and HU values or angiography with contrast injection may provide a comprehensive view and vascular indications of the pelvic mass. By contrast, three-dimensional CTU or magnetic resonance imaging aid in delineating the precise location of the renal mass and its association with the collecting system and renal vessels (2). However, occasionally the distinction between TCC with renal invasion and RCC with renal pelvic extension is difficult, since imaging findings of hypovascular RCC demonstrate location, shape and enhancement that are indistinguishable from TCC. Although significant differences in multiphase CT attenuation have been reported between RCC and TCC, the application of these results may be limited to facilitating the differential diagnosis when the diagnosis is uncertain and avoiding biopsy (11). In cases of uncertain diagnosis, the present study advocates that appropriate ureteroscopy and biopsy should be performed to provide crucial histopathological information as the most important evidence. In certain cases, ureteroscopy cannot be performed due to urethral edema, stricture or obstruction; therefore, frozen section analysis is recommended to determine any required changes to the surgical strategy (4,5,9).

RCC with renal pelvic invasion is hypothesized to occur due to the hollow structure of the renal pelvis. Invasion is easier in the renal pelvis compared with the renal parenchyma when localized RCC originates in the marginal parenchyma surrounding the renal pelvis or when RCC invades the entire kidney. In the present case, RCC did not invade the normal urothelium of the renal pelvis; however, in a number of previous cases, the tumors had expanded into the ureter and bladder (7,9). Therefore, the present study provides fundamental evidence for the following theory: Implantation and/or invasion of the urothelial mucosa followed by intraluminal expansive growth results in the pathogenesis of RCC with pelvic extension (9). Previous cases reporting RCC growth along the urinary tract support the following mechanism of RCC metastasis: Tumor cells may metastasize via intraluminal transit down the urinary tract and invade the distal ureter or bladder (7,9). Although these previous cases provide evidence of a possible mechanism of RCC growth, they also challenge the present tumor node metastasis (TNM) staging system. The case reported in the present study indicates that RCC with invasion of the urinary collecting system should be included in the present TNM staging system. However, the inclusion of the urinary collecting system invasion of RCC in the TNM staging system remains controversial (12,13).

In conclusion, in this study, a rare case of RCC growing into the renal pelvis was presented and the role of ureteroscopy and biopsy in accurate preoperative diagnosis was highlighted. The present study provided fundamental evidence for the pathogenesis of RCC with pelvic extension and challenged the present TNM staging system of RCC. However, whether the invasion of the urinary collecting system is an independent prognostic factor in RCC remains controversial and thus, further studies are required.

## Figures and Tables

**Table I:** Reported cases of RCC growing into the renal pelvis.

Case	Reference, year	Age (years)/gender	CT description	Ureteroscopy or cystoscopy/biopsy	Preoperative diagnosis	Histological diagnosis
1	Munechika *et al* (3), 1990	22/M	Calcified tumor in the lower pole of the right kidney, renal pelvis and proximal ureter	Not performed	TCC	Mixed-type RCC
2	Chen *et al* (6), 1996	62/F	Tumor in the right kidney and renal pelvis	Not performed	Uncertain	RCC
3	Gulati *et al* (7), 2007	67/M	Irregular kidney mass and bladder mass	Cystoscopy revealed a 5-cm mass emanating from the left ureteral orifice/RCC	RCC	Clear-cell RCC
4	Fujita *et al* (8), 2011	43/M	Enhanced tumor in the left kidney, renal pelvis, ureter and bladder	Cystoscopy revealed no obvious tumorous lesion/not performed	RCC	Clear-cell RCC
5	Kitazono *et al* (4), 2011	64/M	Enhanced mass in the left kidney upper pole, renal pelvis and ureter	Not performed	Uncertain	Clear-cell RCC
6	Kitazono *et al* (4), 2011	77/F	Large mass in the right kidney invaded the pelvicaliceal system	Ureteroscopy confirmed pelvicaliceal involvement/not performed	Uncertain	Clear-cell RCC
7	Jeong and Kim (5), 2012	58/F	Enhanced left renal pelvic mass	Cystoscopy was negative for TCC/not performed	TCC	Clear-cell RCC
8	Kakutani *et al* (9), 2013	51/F	Contrasted tumor in the left, kidney renal pelvis, ureter and bladder	Cystoscopy revealed a tumor emanating from the left ureteral orifice/not performed	Renal pelvic tumor	Clear-cell RCC
9	Present study, 2015	51/M	Enhanced right renal pelvic mass	Ureteroscopy confirmed a renal pelvic mass/clear cell RCC	Clear-cell RCC	RCC

RCC, renal cell carcinoma; M, male; F, female; CT, computed tomography; TCC, transitional cell carcinoma.

## References

[1] Jemal A, Siegel R, Xu J, Ward E (2010). Cancer statistics, 2010. CA Cancer J Clin.

[2] Rha SE, Byun JY, Jung SE (2004). The renal sinus: pathologic spectrum and multimodality imaging approach. Radiographics.

[3] Munechika H, Kushihashi T, Gokan T, Hashimoto T, Higaki Y, Ogawa Y (1990). A renal cell carcinoma extending into the renal pelvis simulating transitional cell carcinoma. Urol Radiol.

[4] Kitazono MT, Coakley FV, Naeger DM, Yeh BM, Joe BN, Qayyum A (2011). CT of unusual renal masses invading the pelvicaliceal system: potential mimics of upper tract transitional cell carcinoma. Clin Imaging.

[5] Jeong YB, Kim HJ (2012). Is it transitional cell carcinoma or renal cell carcinoma on computed tomography image?. Urology.

[6] Chen WC, Lee YH, Huang JK (1996). Renal cell carcinoma with renal pelvic extension simulating transitional cell carcinoma: a case report. Zhonghua Yi Xue Za Zhi (Taipei).

[7] Gulati M, Gore JL, Pantuck AJ, Kim Y, Barajas L, Rajfer J (2007). Ureteral tumor thrombus from renal cell carcinoma extending into bladder. Urol Oncol.

[8] Fujita O, Wada K, Yamasaki T, Manabe D, Takeda K, Nakamura S (2011). Renal cell carcinoma with a tumor thrombus in the ureter: a case report. BMC Urol.

[9] Kakutani S, Kume H, Hirano Y, Wakita T, Homma Y (2013). Renal cell carcinoma with intraluminal spread of the entire upper urinary tract. Case Rep Med.

[10] Cowan NC (2012). CT urography for hematuria. Nat Rev Urol.

[11] Bata P, Tarnoki DL, Tarnoki AD (2014). Transitional cell and clear cell renal carcinoma: differentiation of distinct histological types with multiphase CT. Acta Radiol.

[12] Verhoest G, Avakian R, Bensalah K (2009). Urinary collecting system invasion is an independent prognostic factor of organ confined renal cell carcinoma. J Urol.

[13] Waalkes S, Merseburger AS, Herrmann TR (2010). Urinary collecting system invasion is no independent prognostic factor in renal cell carcinoma. World J Urol.

